# Spatial variations in wet-bulb globe temperature during the Bank​ of America Chicago Marathon

**DOI:** 10.1007/s00484-026-03361-8

**Published:** 2026-11-02

**Authors:** Zachary J. Suriano, Joshua Durkee, Michael Quire, George Chiampas

**Affiliations:** 1 Department of Earth, Environmental, and Atmospheric Sciences, Western Kentucky University, Bowling Green, USA; 2 Departments of Emergency Medicine and Orthopaedic Surgery, Northwestern University, Chicago, USA; 3 Chief Medical and Safety Officer, Bank of America Chicago Marathon, Chicago, USA

**Keywords:** WBGT, Heat stress, Micro-scale variation, Marathon, Chicago

## Abstract

Prior work has recommended the use of Wet-Bulb Globe Temperature (WBGT) to inform operational management of marathons. However, there is limited and potentially conflicting results on the spatial extent at which WBGT should be measured to sufficiently capture racecourse variability. The purpose of this study is to assess how WBGT varies spatially and temporally across the Bank of America Chicago Marathon course, and if practically and statistically significant differences exist. WBGT was measured at 60-second intervals and averaged every five minutes at five different locations across the Chicago Marathon course in 2022, 2023, and 2024. Substantial temporal and spatial variability in WBGT and meteorological conditions was observed within and between marathon years. Statistically significant differences in WBGT, and other heat stress metrics, existed between the five weather stations in each of the three marathon years (W-ANOVA, *p* < 0.01), with maximum mean absolute differences during 07:30 − 11:00 of 2.6 °C, but with maximum differences of 9.3 °C at individual timesteps using a rolling intervals framework. Results were consistent between fixed periods of time and when considering only the periods in which runners were present at any given portion of the course. Collectively, the observed differences in WBGT and related meteorological conditions along the Chicago Marathon route were statistically and practically significant for operational management. The use of a multiple-station observational approach is warranted for most appropriately capturing racecourse variability and thus informing decisions related to participant health and safety.

**Supplementary Information:**

The online version contains supplementary material available at https://doi.org/10.1007/s00484-026-03361-8.

## Introduction

Meteorological conditions play a critical role in influencing runner performance and health during a marathon. Whether by absorbed solar radiation or through exposure to ambient air temperatures, warm and sunny meteorological conditions experienced by a marathon participant can increase heat stress and result in negative medical outcomes (Trapasso and Cooper 1989; Cheuvront and Haymes 2001; Roberts 2010; Schroeder et al. 2023). Heat stress can be increased further based on levels of atmospheric moisture, where high dewpoint temperatures and relative humidity, with low wind speeds, may inhibit the transfer of latent heat from participants by limiting the ability for sweat or water on the skin to evaporate (Vihma 2010). Arguably, the Wet-Bulb Globe Temperature (WBGT) is the most used metric for assessing heat stress in the marathon industry and is near exclusively used in athletics in the United States, incorporating air temperature, humidity, wind speed, and solar radiation measurements into a single variable that can be efficiently communicated (Liljegren et al. 2008).

Historically, assessments of meteorological conditions for operational marathon management have been relatively limited in scope. Many race organizers and managers do not account for weather conditions beyond simply monitoring the forecast from a local newscast to assess the potential for hazardous conditions. Others may rely on a nearby established weather station, such as at a local airport, for their near-real-time weather needs. Then there is a relatively smaller subset with the resources and knowhow to conduct their own on-site monitoring. In these cases, observations of WBGT and other conditions are often conducted at or before the start of the race at a single location, typically near the start/finish line, to inform a start/no-start decision and other operational decisions based on established flag levels (Roberts 2010; Cheuvront et al. 2015; Schroeder et al. 2023). Prior research into the efficacy of utilizing a single weather station on-site suggests it offers greater representation of racecourse conditions than that of nearby automated stations (e.g., local airport), but that insignificant differences between stations within the course were observed (Cheuvront et al. 2015, 2023). However, conflicting results are observed by marathon location, where prior studies preemptively assessing the Tokyo Olympic Marathon (Vanos et al. 2018) and the Comrades Ultra-Marathon (Havenga et al. 2024) find meaningful differences in heat stress metrics exist spatially across their respective courses. Given these differences, further analysis into spatial variability in meteorological conditions across a marathon course is warranted.

Prior research finds that spatial differences in land use and land cover, and proximity to large bodies of water, can impact heat stress due to impacts on the meteorological conditions (Wang et al. 2023; Harmay and Choi 2023; Chakraborty et al. 2023; Park et al. 2026). In urban centers, the prevalence of man-made materials (e.g., steel, glass, concrete) decreases the albedo and results in increased temperatures due to increase in the proportion of solar radiation being absorbed at the surface, collectively referred to as an Urban Heat Island (Zhao et al. 2014, 2021; Hsu et al. 2021). The prevalence of large buildings in the urban environment further impact weather though shading at different times of day and at different sun angles (Yi et al. 2024; Middel et al. 2018; Chapman and Thornes 2004; Miao et al. 2020), and through the channeling of wind (Hu et al. 2026; Mittal et al. 2018). Additionally, locations at the boundaries of different landscapes, such as forests, fields, or water, further experience micro-scale variability due to enhanced turbulence and advection. For example, in Chicago, the proximity of the city to Lake Michigan routinely results in lake- and land-breezes (Keeler and Kristovich 2012) which may influence heat stress, complicated by the variability of urban land cover in Chicago that results in compounding variations in heat stress during simulated summer months (Chakraborty et al. 2023).

The Chicago Marathon, one of the largest marathons in the world, has a history of weather impacts that have led to changes in operational management of the event with time. Changes largely stem from the 2007 marathon in which race organizers made the decision mid-race to cancel the event due to extreme conditions of air temperatures exceeding 30 °C with dewpoint temperatures of 20 °C (Başdere et al. 2014; Durkee et al. 2026). Since then, the holistic Chicago Model approach has been utilized to streamline communication, issue alerts, and enhance planning efforts (Hanken et al. 2016; Chiampas and Jaworski 2009). Starting in 2022, Chicago Event Management has partnered with the Western Kentucky University Disaster Science Operations Center to provide on-site weather support, sampling, and communications for the Chicago Marathon and similar events (Durkee et al. 2026). Given the history of weather-related challenges and current weather support and infrastructure for the Chicago Marathon, and its relatively unique microclimatological conditions, Chicago makes an ideal candidate to evaluate for the presence of spatial variability in meteorological conditions during marathons.

Using a micro-network of five weather stations, the objective of this study is to quantify the differences in WBGT and related meteorological conditions spatially across the racecourse of the 2022, 2023, and 2024 Bank of America Chicago Marathon in Chicago, IL, United States. It is hypothesized that statistically and practically significant differences in WBGT and related variables will exist during all three marathons years sampled. Such a result would inform race management strategy at the Chicago Marathon and could be considered for adoption by other marathon organizers.

## Methods

### Study area

**Fig. 1:**
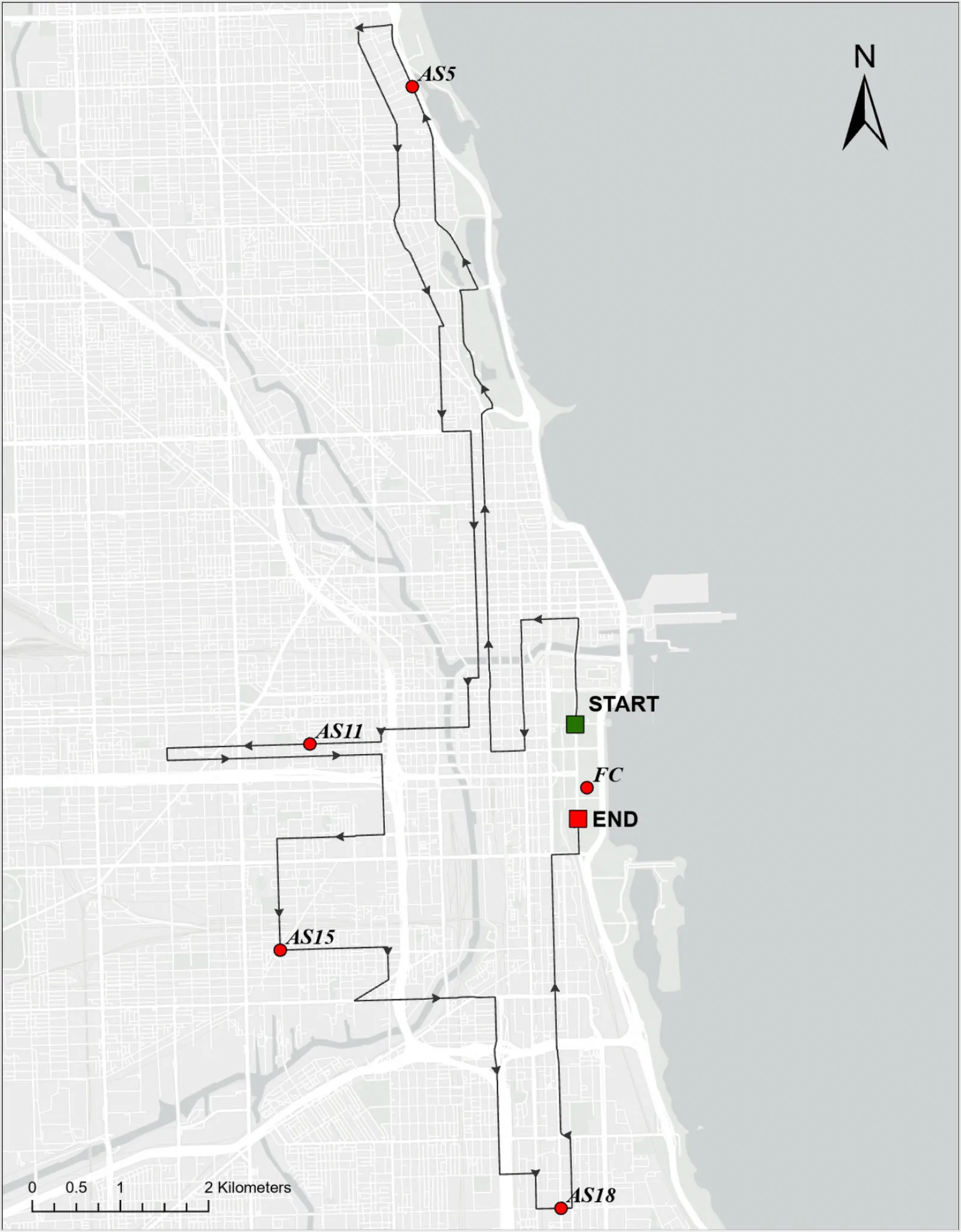
Chicago Marathon course with locations of in-situ weather monitoring sites at four aid stations, the forward command operations center (AS5, AS11, AS15, AS18, and FC), and the start and end lines marked

The study was conducted at the 2022–2024 Chicago Marathons on October 9, 2022, October 8, 2023, and October 13, 2024. The course begins at Grant Park and traverses the North, West, and South Sides of downtown Chicago (Fig. 1). The first approximately four miles of the course snake through the dense central corridor of Chicago, predominantly in a north/south orientation, surrounded by skyscrapers within the Loop and River North neighborhoods. From mile 5 to 11, the course runs north and returns south through Lincoln Park, Old Town, Wrigleyville and east Lakeview neighborhoods along Lake Michigan (to the east). This northern section is predominantly residential with relatively higher tree cover. Miles 13–18 traverse the West side of the course, including neighborhoods of West Loop, Greektown, and Little Italy. The West side of the course is generally more open, with a higher frequency of parking lots and low-rise buildings. Following mile 18, the course travels south and east, progressing through the Pilsen, Chinatown, and Park Boulevard regions. This stretch is largely industrial and low-rise residential, with limited tree cover at times. Mile marker 23 is approximately the southernmost point of the course, following which the course turns north back through portions of Douglas and returns to Grant Park (Fig. 1). The race typically admits between 40,000 and 50,000 participants and has recently held the men’s, women’s mixed, and wheelchair world records. As of October 2026, both the wheelchair and women’s mixed records were held by the Chicago Marathon.

To enhance understanding of the underlying larger-scale meteorological conditions occurring during each marathon, brief overviews are presented. On October 9, 2022, large-scale flow over the greater Chicago area was relatively zonal, associated with a 1027 hPa High over the Mid-South United States, and a 1000 hPa Low over northern Quebec with a trailing cold front stretching across the Upper Peninsula of Michigan. The synoptic-scale conditions led to flow generally from the southwest with clear skies throughout the majority of the marathon. On October 8, 2023, flow was largely from the west-north-west over northern Illinois driven by a 992 hPa Low over eastern Quebec and a relatively tight pressure gradient over the Great Lakes region. Sky conditions were cloudy throughout the region, with reported ceilings between 2.3 and 5.1 km. There were multiple observations of very light drizzle around 2:10pm local time but otherwise conditions were reported as dry. On October 13, 2024, a relatively weak 1007 hPa Low was centered over northeastern Ohio, placing Chicago on the backside of the Low. This synoptic configuration resulted in increasing winds out of the west-north-west with scattered cloud cover and no reported precipitation. The day prior to the 2024 marathon, light precipitation was observed across Chicago.

### Data collection

Calibrated Kestrel 5400 Heat Stress Tracker devices were mounted onto Vicitiv NT70 Tripods with Kestrel Wind Vane attachments at a height of 1.5 m, corresponding to average runner chest height. Henceforth, this setup will be referred to as the weather stations. Weather stations were deployed at Aid Stations (AS) 5, 11, 15, and 18 along the course, and at the Forward Command (FC), where they were placed on the race surface (e.g., concrete and/or asphalt; Fig. 1; Table 1). The decision to place the weather stations on the running surface, as opposed to the nearby natural surfaces (i.e., grass, dirt), was driven by the need to ensure the weather stations were most representative of conditions experienced by the marathon participants. Visuals of the station step and physical surrounds are provided as Supplemental Material (Supplemental Figs. 1–5).

AS5 is located on inner Lake Shore Drive with roughly 40 m of divided highway to its immediate east before approximately 350 m worth of mixed landcover associated with Lincoln Park and a thin zone of Belmont Harbor. To the west, opposite the AS5 weather station on inner Lake Shore Drive, there are multi-story buildings extending upwards of 15 stories tall with a thin line of tree cover immediate in front of the buildings (approximately 5 stories tall; Table 1). AS11 is located on Adams Street, southeast of Skinner Park (open fields) with a parking lot adjacent to the south and at least 15–20 m of horizontal clearance until tree cover not more than 5 m in vertical extent Approximately 300 m south of AS11 is a major divided interstate (I-290) and multiple rail tracks. AS15, in the southwest portion of the course, is located on Loomis Street, situated with approximately 100 m of parking lot to its east and two-story residential buildings approximately 60 m to the north. Prior to 2024, the land to the northwest and west was an open, unirrigated, grassy field. In 2024, this adjacent land was redeveloped to now be a series of artificial turf sporting fields. AS18 represents the southern-most point of the course near the intersection of Michigan Ave and 35th St (Fig. 1; Table 1). To the southwest of the station is the approximately five-story Chicago Public Safety Headquarters, and to the northwest a single-story building. The FC station is located within Grant Park, roughly halfway between the start and finish lines, less than 100 m east of the central Buckingham Fountain and approximately 150 m west of Lake Michigan.

**Table 1:** Weather station site characteristics and arrival and departure of first and last runners, respectively, rounded to the nearest 5-minute interval

Weather​ Station	AS5	AS11	AS15	AS18	FC
Location	Inner Lake Shore Drive	Adams Street	Loomis Street	Michigan Ave	Grant Park
Approximate Distance into Course	7 miles (11 km)	15 miles (24 km)	19 miles (30.4 km)	23 miles (36.8 km)	Start / Finish
Elevation	179.5 m	180.3 m	179.6 m	180.3 m	181.9 m
Distance to Lake Michigan	400 m	4,000 m	3,600 m	1,600 m	150 m
Sky View	Unobstructed	Unobstructed	Unobstructed	Unobstructed	Unobstructed
First Runner 2022	08:05	08:40	09:05	09:15	07:30
Last Runner 2022	11:00	12:40	13:40	15:00	15:30
First Runner 2023	08:05	08:40	09:05	09:15	07:30
Last Runner 2023	11:00	12:40	13:45	14:45	15:30
First Runner 2024	08:00	08:40	08:55	09:15	07:30
Last Runner 2024	11:00	12:40	13:45	14:45	15:30

These station locations were selected based on multiple factors, first and foremost spatial alignment with existing medical staff at the ASs. Co-occurrence with these locations aids in logistical simplicity and staff safety. Secondly, the five stations represent different primary directions of the course (N, W, SW, S, central) as to assess a diversity of locations while also capturing different segments of the course with respect to the 26.2-mile race. It is feasible that the selection of different AS locations may yield different results, however these serve as a justifiable distribution in capturing the footprint of the course. Third, these locations had reasonable access before, during, and after the marathon, allowing sufficient time for each station to be setup and acclimated prior to the race beginning. Additional considerations included ensuring sufficient space to place the station to limit immediate impacts from overhead cover (e.g., not placing the station under a tree), avoiding locations which experienced shadow rollover (i.e., initially in the sun but with time and changing sun angles, a pole or tree casts a shadow onto the station), not positioning next to a secondary heat source such as a building or reflective glass, and avoiding direct impediment of the runners and/or medical staff.

The Kestrel weather stations were selected based on use in similar research designs, where their simplicity yet high accuracy in measuring WBGT was advantageous (Hall et al. 2023; Clark and Konrad 2024). Stated accuracies of the Kestrel stations are as follows: outdoor WBGT (0.7 °C), ambient temperature (0.5 °C), globe temperature (1.4 °C) and relative humidity (2%; Kestrel Instruments 2026). To ensure reliability between weather stations, the devices were calibrated following manufacture guidance in advance of the marathon and further allotted at least 20 min of acclimatization prior to data collection the day of the marathons. Side-by-side comparison was conducted prior to the marathon during a mock 1-hour staging where stations were further compared against a Davis Vantage Pro2 station to ensure consistent, accurate measurements between stations. Data collection rate was set at 60 s and averaged over 5-minute periods for statistical analysis. This was deemed appropriate given the sensitivity of the weather stations to minor environmental changes (e.g., passing clouds, wind changes), and thus reporting instantaneous values may not appropriately reflect the average running environment during the sample. Data were entered manually using the Survey 123 mobile App by observers initially for operational and reporting purposes, where protocols entail the entered observations are confirmed by a secondary observer prior to submission to limit potential for typographical errors. Data were additionally automatically logged on the internal data logger and cross-referenced for verification purposes. Flag levels used were prescribed by the Chicago Event Management (Durkee et al. 2026), where green, yellow, red, and black flag levels were set as WBGT values of at least 10 °C, 18 °C, 23 °C, and 28 °C, respectively. Operationally, green flag conditions entail an Event Alert System (EAS) response towards standard monitoring, compared to no action required for white/no flag below 10 °C, increased monitoring and elevated medical staff alerts for yellow flag, delayed start and further medical staff alert and deployment for red flag, and a response towards cancelation, postponement, and/or suspension of the event for black flag (Durkee et al. 2026).

WBGT data were compared against gridded estimates of WBGT derived from ERA5-Land variables of temperature, dewpoint temperature, pressure, wind speed, and downward shortwave radiation at its native 9 km spatial and hourly temporal resolutions (Muñoz-Sabater et al. 2021; Spangler et al. 2022). ERA5-Land grid cells aligning spatially with the geographic locations of the five weather stations based on a centroid method were extracted and WBGT calculated using the formulations developed by Liljegren et al. (2008) for four individual hours (08:00–11:00 local). In this case, three grid cells represent the five stations, with AS11 and AS15, and AS5 and FC, being geographically matched with the same 9 km grid cell. Means and differences were presented and compared against those of the weather stations. Further, while the focus of the study is WBGT, based on its use by World Athletics, the international governing body for the sport of athletics, complementary analyses were performed based on collected observations with two additional metrics: Heat Index and Humidex. The Heat Index is an apparent temperature which accounts for humidity and air temperature, providing a ‘feels-like’ temperature which can be quickly communicated and interpreted, and was calculated following Rothfusz (1990; Steadman 1979). Humidex is a similar index used by the Meteorological Service of Canada to estimate the physiologic impact of heat on people (Masterton and Richardson 1979; Li et al. 2023) and was calculated based on the formulations presented in Spangler et al. (2022).

### Statistical analysis

Descriptive statistics, inclusive of means and standard deviations, of WBGT, air temperature, and dewpoint temperature were calculated at the five weather stations during the three marathons. Observations were analyzed two separate ways: (1) “Fixed Time Intervals” – analysis over identical time periods in which all five stations reported consistently, and (2) “Rolling Intervals” – analysis over only the respective periods in which runners were physically present at the individual stations. For the Fixed Time Interval analysis framework, observations were assessed from 7:30 − 11:00am (CDT) on each race day, coinciding with the start of the race. Analysis of all five stations after 11:00am was not possible given once the final runner passes, course logistics mandates ASs and weather reporting shut down to reopen that portion of the course to the public (i.e., AS5 ceased operations at ~ 11:00am). While this framework inhibits understanding meteorological conditions during the later portions of the race, the sample allowed for identical periods of time to be compared, thus for the isolation of spatial differences in meteorological conditions.

The Rolling Interval analysis framework, akin to the approach used by Havenga et al. (2024), provides a different, but complementary perspective in that the periods of time for which stations are assessed are not the same. Instead, analyzed observations are based on if runners were physically present at the station, with the first observation corresponding to when the lead runner reached the station and the last observation corresponding to when the last runner left the station, making for a rolling period to which conditions of runner exposure were assessed. This allows for a more tailored assessment of conditions experienced by runners across the course but does introduce the potential for the descriptive statistics to be based on a different number of samples for each station – an acceptable outcome given the benefits of the framework. Further, under this framework, stations located farther into the course are sampled later in the day, thus differences observed under the Rolling Interval framework would include both spatial and diurnal effects.

To assess the potential for spatial variations in WBGTs, a Welch’s ANOVA was conducted with a Post Hoc Games-Howell test for each marathon and for each analysis framework. The null hypothesis was that there were not significant differences in WBGT across the five stations for either framework, based on an alpha level of 0.05. All statistical testing was performed in SPSS version 31.0.0. Similar analyses were performed with other variables such as air temperature and dewpoint temperature, and Heat Index and Humidex. It should be noted that slight differences in the number of samples exist between marathon years based on isolated instances of missing data and the potential for a different number of periods to exist per station if the last runner reaches a particular station at a later or earlier time. The number of samples are reflected in the statistical summaries within the results.

## Results

### Fixed time intervals framework

**Fig. 2:**
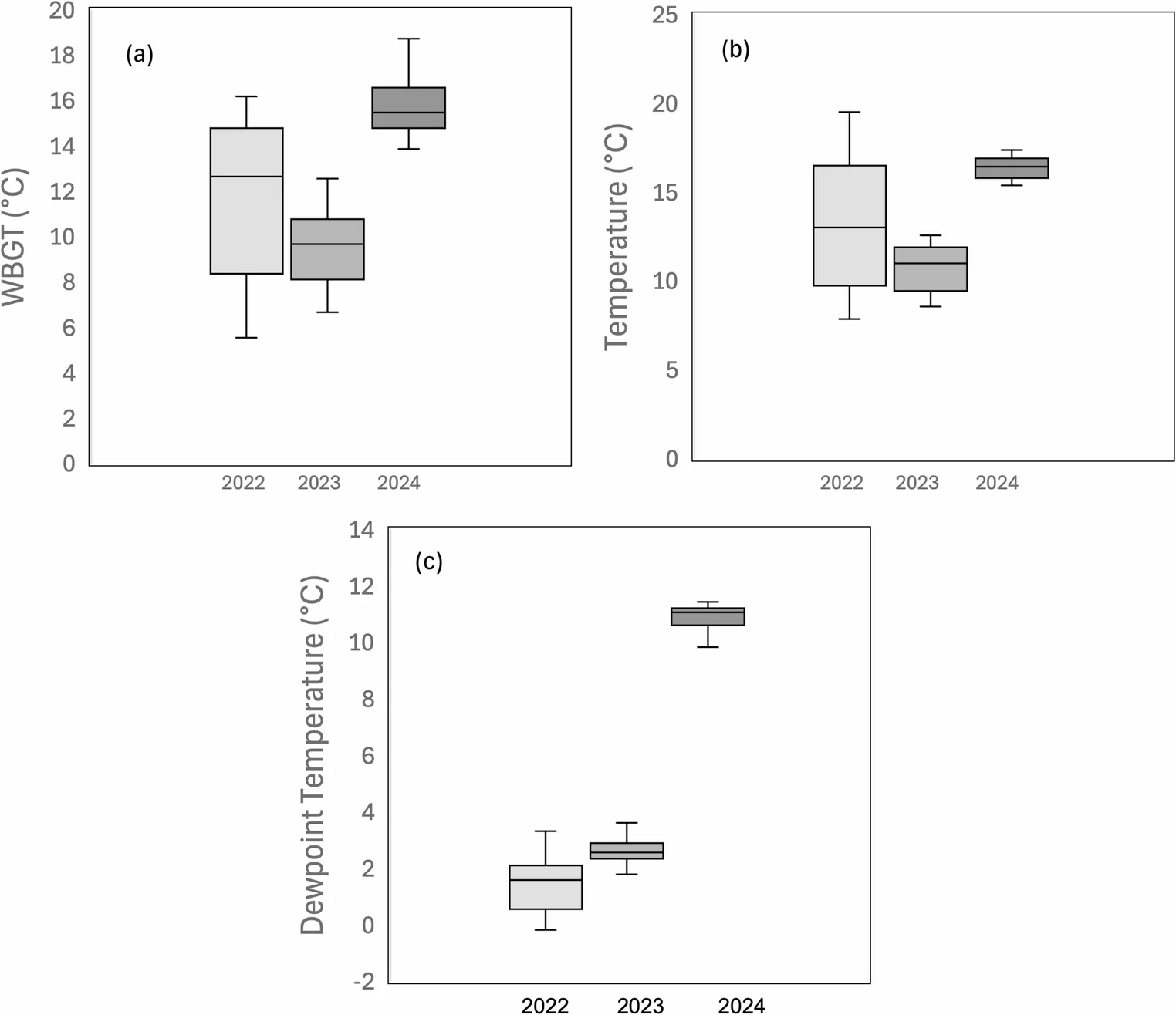
Meteorological conditions of (**a**) WBGT, (**b**) air temperature, and (**c**) dewpoint temperature, in °C, during the 2022 (light gray), 2023 (medium gray), and 2024 (dark gray) Chicago Marathons during the sample period of 7:30 − 11:00am (CDT). Conditions were averaged across all five stations for each timestep, per year, such that whiskers represent the range of spatially averaged conditions

Of the three marathons, the 2023 marathon had the coolest WBGT aggregated across all five weather stations, averaging 9.48 °C, compared to the 2022 and 2024 marathon averages of 11.45 °C and 15.66 °C, respectively during the 07:30 − 11:00am periods (Fig. 2a). Similar differences were noted for air temperature with 2022, 2023, and 2024 marathons exhibiting averages of 13.05 °C, 10.78 °C, and 16.39 °C, respectively (Fig. 2b). Dewpoint temperatures were similar in 2022 and 2023, averaging 1.41 and 2.73 °C, respectively, while substantially higher dewpoint temperatures of 10.90 °C were observed in 2024 (Fig. 2c).

**Fig. 3:**
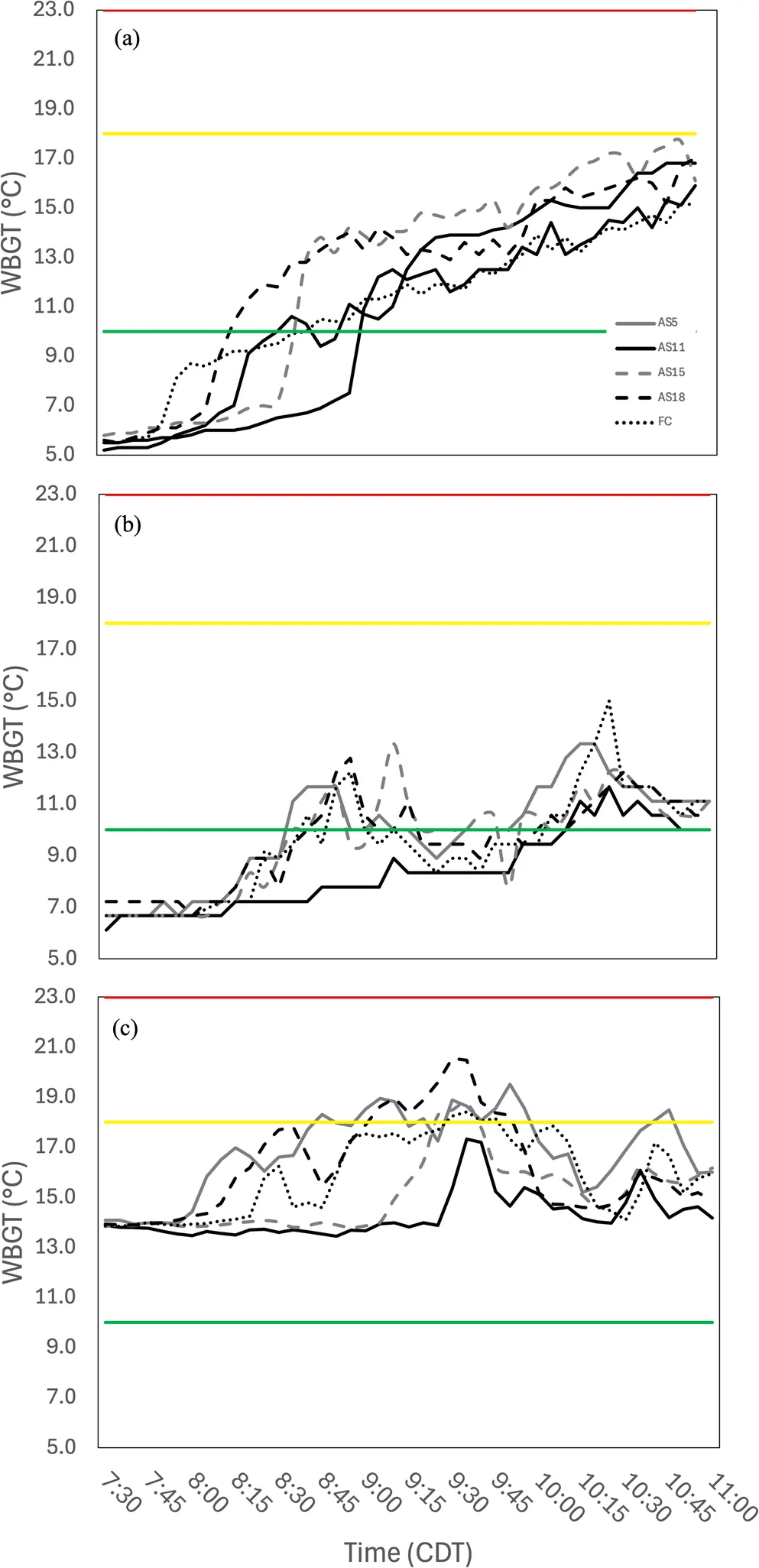
Time series of WBGT at AS5 (solid gray), AS11 (solid black), AS15 (dashed gray), AS18 (dashed black), and FC (dotted black) weather stations in °C from 7:30 − 11:00 am (CDT) for the (**a**) 2022, (**b**) 2023, and (**c**) 2024 Chicago Marathons. Flag levels are marked. The legend in panel (**a**) applies to all three panels

Substantial variability in race-day WBGT and related meteorological variables was observed temporally and spatially (Table 2; Fig. 3). During each marathon, WBGT increased with time at all weather stations from 07:30 − 11:00am, in some cases by over 10 °C, however not necessarily linearly (Fig. 3). For example, WBGT increased over the first two hours of the marathon in 2024 at multiple weather stations before decreasing for the remaining hours of the sample for a net, but ultimately modest, increase with time (Fig. 3c). Air temperatures steadily increased during all three marathons while dewpoint temperatures remained steady (in 2023 and 2024) or increased (in 2022).

**Table 2:** Descriptive statistics of 07:30 − 11:00am WBGT, air temperature (Ta), and dewpoint temperature (Td) at the five weather station locations in 2022–2024 marathons

	FC	AS5	AS11	AS15	AS18
2022					
Mean WBGT (°C)	11.08	11.51	10.25	12.40	12.39
Stdev WBGT (°C)	2.78	3.90	3.72	4.35	3.53
Mean Ta (°C)	11.05	13.15	12.95	13.80	14.32
Stdev Ta (°C)	2.18	3.82	4.55	4.07	4.15
Mean Td (°C)	1.18	1.60	0.93	1.69	1.65
Stdev Td (°C)	0.89	1.39	0.99	1.33	1.11
2023					
Mean WBGT	9.47	9.90	8.41	9.61	9.61
Stdev WBGT	2.09	2.04	1.58	2.00	1.72
Mean Ta	10.31	11.25	10.31	11.08	10.93
Stdev Ta	1.35	1.64	1.30	1.66	1.22
Mean Td	2.79	2.86	2.24	2.39	2.42
Stdev Td	0.55	0.57	0.44	0.70	0.50
2024					
Mean WBGT	15.87	16.85	14.27	15.09	16.25
Stdev WBGT	1.59	1.61	0.91	1.39	1.95
Mean Ta	16.54	16.33	15.78	16.64	16.67
Stdev Ta	0.73	0.71	0.59	0.97	0.65
Mean Td	11.17	11.01	10.59	10.71	11.00
Stdev Td	0.61	0.48	0.35	0.40	0.46

Welch ANOVA testing rejects the null hypotheses for all three years, indicating statistically significant differences in WBGT exist between the weather stations across the marathon course (2022: F = 2.574, (4,205), *p* = 0.039, Welch: 2.480, *p* = 0.049; 2023: F = 4.285, (4,210), *p* = 0.002, Welch: 5.43332, *p* < 0.001; 2024: F = 18.722, (4,210), *p* < 0.001, Welch: 27.021, *p* < 0.001). Games-Howell Post Hoc tests in 2022 indicate significant differences of approximately − 2.14 °C between AS11 and AS18. In 2023, Games-Howell Post Hoc tests indicate significant differences in WBGT between AS11 and FC (-1.06 °C), AS5 (-1.49 °C), AS15 (-1.20 °C), and AS18 (-1.20 °C). Finally, in 2024, Games-Howell Post Hoc tests indicate significant differences in WBGT between FC and AS5 (-0.98 °C) and AS11 (1.60 °C), between AS5 and AS11 (2.58 °C) and AS15 (1.76 °C), between AS11 and AS15 (-0.82 °C) and AS18 (-1.98 °C), and between AS15 and AS18 (-1.15 °C).

Similar results were obtained when assessing spatial variability in Heat Index and Humidex conditions (Supplemental Fig. 8). For all three marathon years, statistically significant differences in both variables were observed. For Heat Index, maximum differences between stations for concurrent time steps were 8.5 °C over the 07:30 − 11:00am time period (2022 (F = 4.276, (4,210), *p* = 0.002, Welch: 7.418; 2023 (F = 4.129, (4,210), *p* = 0.003, Welch: 4.105); 2024 (F = 12.046, (4,210), *p* < 0.001, Welch: 18.478)). For Humidex, maximum station differences were as large as 8.0 (2022 (F = 4.189, 2(205), *p* = 0.003, Welch: 7.223); 2023 (F = 3.825, (4,210), *p* = 0.005, Welch: 4.019); 2024 (F = 14.276, 2(210), *p* < 0.001, Welch: 22.176). ANOVA testing was also conducted to assess if significant differences in air temperature and dewpoint temperature were observed spatially within marathon years. In all marathons, air temperatures significantly differed spatially (2022: F = 4.399, (4,205), *p* = 0.002; 2023: F = 3.583, (4,210), *p* = 0.007; 2024: F = 10.469, (4,210), *p* < 0.001). Similarly, differences in dewpoint temperatures were observed between stations (2022: F = 3.594, (4,205), *p* = 0.007; 2023: F = 9.551, (4,210), *p* < 0.001; 2024: F = 11.356, (4,210), *p* < 0.001).

Comparing the station WBGT results to gridded estimates derived from ERA5-Land, the reanalysis product exhibits an average cool bias relative to station observations (Table 3). The cool bias averaged 1.7 °C across all stations and marathon years, with 2022 exhibiting the largest bias (3.1 °C) compared to 2023 with the smallest bias (0.7 °C). Spatially, the grid cell aligned with the most interior station relative to Lake Michigan (AS11) exhibited the smallest bias at just 0.7 °C, compared to 1.9–2.2 °C for the other cells (Table 3).

**Table 3:** ERA5-land WBGT conditions in °C and differences between observed and ERA5-Land derived WBGT conditions in parentheses by AS and marathon year

	41.9°*N*, 87.6°W AS5	41.9°*N*, 87.7°W AS11	41.9°*N*, 87.7°W AS15	41.8°*N*, 87.6°W AS18	41.9°*N*, 87.6°W FC
2022					
8:00	6.4 (-0.4)	5.8 (0.0)	5.8 (0.5)	6.3 (0.1)	6.4 (2.3)
9:00	8.4 (2.3)	8.2 (2.7)	8.2 (5.7)	8.4 (4.9)	8.4 (2.9)
10:00	10.3 (4.6)	10.4 (2.7)	10.4 (5.4)	10.3 (5.0)	10.3 (3.6)
11:00	12.2 (4.6)	12.5 (3.4)	12.5 (3.6)	12.2 (4.8)	12.2 (2.9)
2023					
8:00	6.6 (0.6)	6.2 (0.5)	6.2 (0.5)	6.5 (0.2)	6.6 (0.2)
9:00	7.7 (2.3)	7.5 (0.2)	7.5 (1.9)	7.6 (3.0)	7.7 (2.3)
10:00	9.6 (2.1)	9.6 (-0.2)	9.6 (0.9)	9.5 (0.5)	9.6 (-0.2)
11:00	10.7 (0.4)	10.9 (-0.9)	10.9 (0.2)	10.7 (-0.2)	10.7 (0.4)
2024					
8:00	13.8 (0.7)	13.5 (-0.1)	13.5 (0.3)	13.9 (0.3)	13.8 (0.2)
9:00	13.9 (4.6)	13.7 (-0.1)	13.7 (0.1)	14.0 (3.9)	13.9 (3.6)
10:00	13.8 (3.3)	13.8 (1.4)	13.8 (2.0)	14.0 (1.2)	13.9 (3.7)
11:00	15.0 (1.0)	15.1 (-0.9)	15.1 (1.1)	15.1 (-0.2)	15.0 (0.9)
Average Difference	2.2	0.7	1.9	1.9	1.9

### Rolling interval framework

**Fig. 4:**
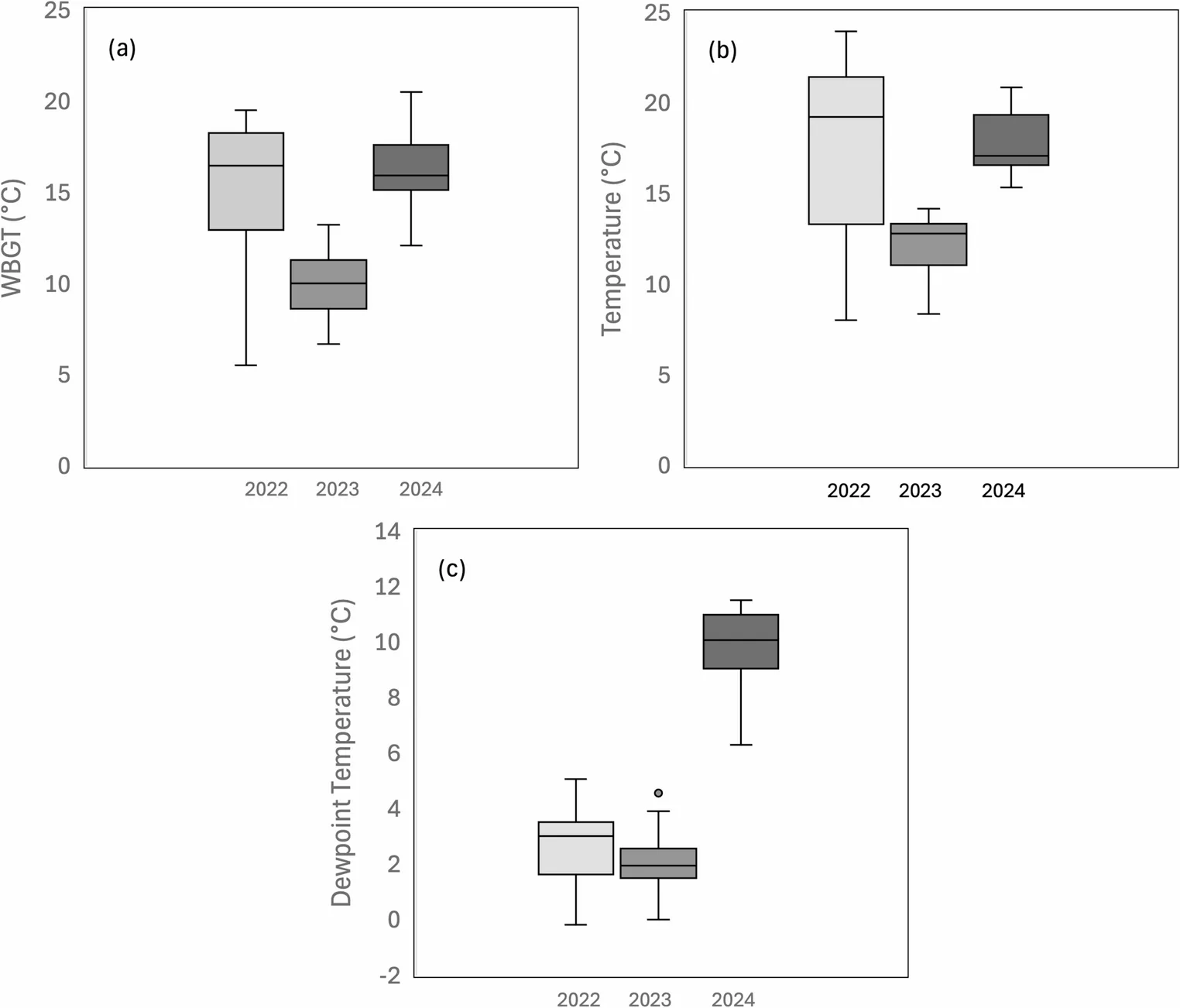
Meteorological conditions of (**a**) WBGT, (**b**) air temperature, and (**c**) dewpoint temperature, in °C, during the 2022 (light gray), 2023 (medium gray), and 2024 (dark gray) Chicago Marathons using the rolling interval framework in which observations are analyzed for the period between which the first and last-place runners passed the respective stations

Similar to the Fixed Time Intervals framework, the 2023 marathon had the coolest WBGT aggregated across all five weather stations for the Rolling Interval framework, averaging 9.93 °C, compared to the 2022 and 2024 marathon averages of 14.88 °C and 16.26 °C, respectively (Fig. 4a). Of note is the increase in WBGT between frameworks, with the Rolling Interval exhibiting higher WBGTs. Similar differences were noted for air temperature with 2022, 2023, and 2024 marathons exhibiting averages of 17.32 °C, 12.06 °C, and 17.58 °C, respectively (Fig. 4b). Dewpoint temperatures were similar in 2022 and 2023, averaging 2.56 and 2.02 °C, respectively, while substantially higher dewpoint temperatures of 9.84 °C were observed in 2024 (Fig. 4c).

**Fig. 5:**
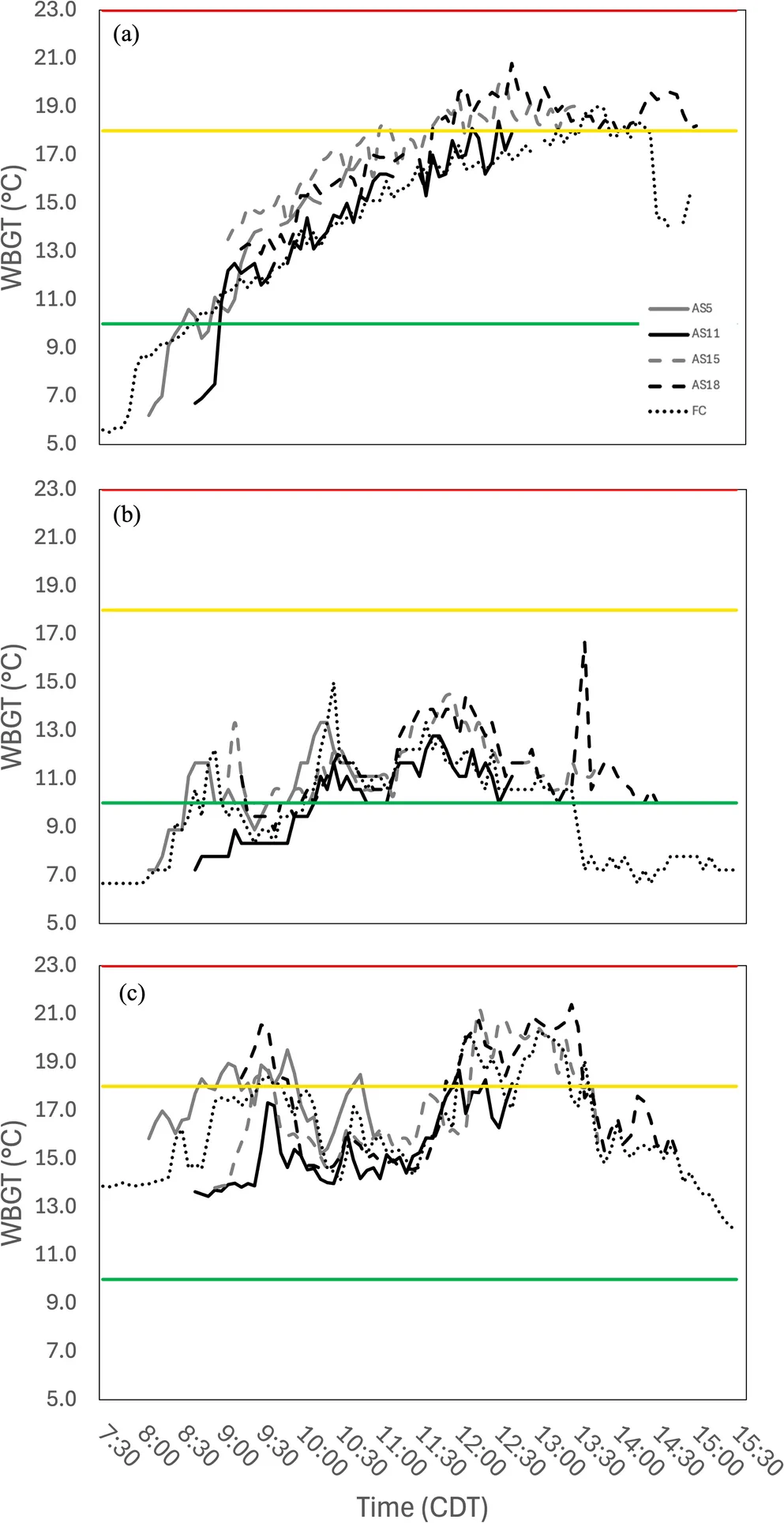
Time series of WBGT at AS5 (solid gray), AS11 (solid black), AS15 (dashed gray), AS18 (dashed black), and FC (dotted black) weather stations in °C for the (**a**) 2022, (**b**) 2023, and (**c**) 2024 Chicago Marathons for only the respective time periods between which the first and last-place runners passed for individual stations (rolling intervals). Flag levels are marked. The legend in panel (**a**) applies to all three panels

Moreso than with the Fixed Time Interval framework, substantial variability was observed in race-day WBGT and related meteorological variables temporally and spatially under the Rolling Intervals framework (Table 4; Fig. 5). During the 2024 marathon, a bi-modal distribution in WBGT was evident across the five stations, with respective peaks corresponding to approximately 9:30am and between 12:00–1:30pm, with minima at the respective beginning and end periods, and between approximately 10:30 − 11:30am (Fig. 5c). In comparison, in 2022 WBGT steadily increased throughout the race at all stations before a rapid decrease in WBGT at the FC station at approximately 2:00pm (CDT). A rapid decrease in FC WBGT was also apparent in 2023, albeit occurring slightly earlier in the day at 1:30pm (CDT). Air temperatures steadily increased during all three marathons while dewpoint temperatures increased (in 2022) or decreased (in 2023 and 2024; see Supplemental Material).

**Table 4:** Descriptive statistics of WBGT, air temperature (Ta), and dewpoint temperature (Td) at the five weather station locations in 2022–2024 marathons during the rolling intervals periods

	FC	AS5	AS11	AS15	AS18
2022					
Mean WBGT (°C)	14.10	12.35	14.19	17.30	17.43
Stdev WBGT (°C)	3.59	3.13	3.01	1.69	2.11
Mean Ta (°C)	15.78	14.22	17.62	19.91	20.40
Stdev Ta (°C)	5.06	3.22	4.04	3.39	2.63
Mean Td (°C)	2.48	1.98	2.10	3.17	3.35
Stdev Td (°C)	1.52	1.20	1.09	1.10	1.12
2023					
Mean WBGT (°C)	9.50	10.49	10.10	11.55	11.45
Stdev WBGT (°C)	2.05	1.51	1.55	1.18	1.57
Mean Ta (°C)	11.58	11.64	11.79	13.03	13.25
Stdev Ta (°C)	1.46	1.23	1.16	1.22	1.36
Mean Td (°C)	2.37	2.90	1.87	1.57	1.58
Stdev Td (°C)	1.21	0.60	0.70	0.97	0.63
2024					
Mean WBGT (°C)	16.18	17.39	15.39	17.19	17.37
Stdev WBGT (°C)	2.02	1.12	1.50	2.03	2.25
Mean Ta (°C)	17.41	16.48	16.50	18.63	18.55
Stdev Ta (°C)	1.49	0.67	0.81	1.63	1.60
Mean Td (°C)	9.97	10.99	10.17	9.98	9.99
Stdev Td (°C)	1.32	0.53	0.49	0.76	1.04

Welch ANOVA testing rejects the null hypotheses for all three years, indicating statistically significant differences in WBGT exhibit between the weather stations across the marathon course when considering only the periods in which runners were present at the stations (2022: F = 30.370, (4,282), *p* < 0.001, Welch: 34.797; 2023: F = 21.540, (4,302), *p* < 0.001, Welch: 22.483; 2024: F = 11.620, (4,303), *p* < 0.001, Welch: 16.433). Games-Howell tests in 2022 indicate significant differences between FC and AS15 (-3.20 °C) and AS18 (-3.33 °C), between AS5 and AS15 (-4.95 °C), and AS18 (-5.08 °C), and between AS11 and AS15 (-3.11 °C) and AS18 (-3.24 °C). In 2023, Games-Howell tests indicate significant differences in WBGT of -0.99 °C between FC and AS5, -2.05 °C between FC and AS15, and − 1.95 °C between FC and AS18. Further, AS5 WBGT was significantly less than AS15 and AS18 by -1.06 and − 0.96 °C, respectively, while AS11 WBGT was also significantly less than AS15 (-1.45 °C) and AS18 (-1.35 °C). Finally, in 2024, Games-Howell tests indicate significant differences in WBGT between FC and three of the other four stations (AS5: -1.21 °C; AS15: -1.01 °C; AS18: -1.19 °C) and between AS11 and three of the other four stations (AS5: -2.00 °C; AS15: -1.80 °C; AS18: -1.98 °C).

ANOVA testing was also conducted to assess if significant differences in air temperature and dewpoint temperature were observed spatially within marathon years. In all marathons, air temperatures significantly differed spatially (2022: F = 24.97, (4,294), *p* < 0.001; 2023: F = 23.25, (4,290), *p* < 0.001; 2024: F = 29.94, (4,303), *p* < 0.001). Similarly, differences in dewpoint temperatures were observed between stations (2022: F = 12.88, (4,294), *p* < 0.001; 2023: F = 26.51, (4,290), *p* < 0.001; 2024: F = 7.41, (4,303), *p* < 0.001).

## Discussion

Analysis of weather station data show significant variability of heat stress exposure across the course during all three sampled marathons and within both analysis frameworks, with significant differences in WBGT observed. Such results persist when assessing other environmental indexes, including Heat Index and Humidex, which further support a physical mechanism and not a function of a single index. Here, similar results were evident between the Fixed Intervals and Rolling Intervals frameworks, where significant differences were apparent between stations in all years, however generally the WBGTs for the Rolling Intervals framework were larger. This is hypothesized to be due to the extension of sampling into the late morning and afternoon hours of the day where there is the potential for increasing temperature and solar radiation inputs within the diurnal cycle. This is similar to prior work which documented increases in WBGT conditions in race segments of an ultra-marathon as time progressed later into the day (Havenga et al. 2024), such that relatively slower runners may experience progressively riskier WBGT conditions with time than the relatively faster runners. When considering the Fixed Intervals framework, the mean multi-hour differences in WBGT between weather stations over the sample period exceeded 2.5 °C. However, when considering individual times during the race, differences between WBGT at the weather stations were as high as 6.9 °C. In 2024, 35% of time steps exhibited different flag levels. When using the Rolling Intervals framework, the maximum concurrent difference in WBGT reached 9.3 °C. Such differences are not just statistically significant, but practically significant as it relates to participant performance and safety, as the differences in flag levels were observed between some stations at the same time steps (e.g., yellow vs. green); in 2024, 30% of time steps were at different flag levels, compared to 0% in 2023, and 38% in 2022. This implies that utilization of a single weather station does not capture all the variance in WBGT for the entire course (e.g., Vanos et al. 2018; Havenga et al. 2024) and contradicts previous research concluding multiple stations did not yield significantly different results (Cheuvront et al. 2023). The persistence of significant differences in WBGT for stations across three marathon years, each with different environmental conditions, suggests this result is not a function of the base meteorological conditions. Instead, the contrast from prior research may be due, in-part, to (a) the micro-scale variances in atmospheric conditions along the course routes for the two marathons, and/or (b) in association with differences in sampling periods and design between the studies. There are substantial differences in land cover and urbanization characteristics between the Chicago and Boston marathon courses, with nearly all of the Chicago Marathon occurring within the urban center of the city, compared to Boston which stretches out linearly through more suburban locations (Cheuvront et al. 2023; Maffetone et al. 2017). As such, well documented micro-scale variations in urban environments increase the sensitivity of weather conditions to specific locations and may directly affect WBGT (Chakraborty et al. 2023; MacLean et al. 2021; Wang et al. 2020).

Further, the experimental design from previous research (Cheuvront et al. 2023) measured WBGT over natural (dirt) surfaces and calculated differences in WBGT at hourly temporal resolutions. To more accurately indicate the WBGT experienced by runners, WBGT was measured, here, on the same surface as the runners would experience (i.e., asphalt and/or concrete). Measuring over dirt would not represent the marathon environment, decreasing the sensitivity of WBGT to meaningful differences in temperature caused by albedo and shortwave radiation absorption and subsequent emission over dirt versus asphalt/concrete. Further, calculating at five-minute temporal scales aligns with standard meteorological observation practices for research- and operational-grade applications (Brock et al. 1995; Frenne et al. 2024; Martens et al. 2017), allowing for more accurate representation of meteorological, and thus WBGT, conditions. The use of hourly temporal scales (Cheuvront et al. 2015, 2023) would overly smooth important temporal variations. The use of on-site WBGT measurement is likely still an improvement over WBGT estimates from adjacent regional weather stations (e.g., airports; Cheuvront et al. 2015), and is supported by evidence in the Supplemental Material (Supplemental Figs. 6 and 7) which indicates air and dewpoint temperatures were consistently lower at the nearby Chicago Midway Airport (41.7856°N, 87.7527°W) than across the five weather stations, at times by over 6.0 °C. The evidence presented here indicates sub-course variability is present and warrants documenting. These added datapoints inform operational procedures and race management (Durkee et al. 2026), such as in strategically staffing medical personnel at aid stations with enhanced historical and/or forecasted heat stress, enhancing situational awareness for intra-course risks through weather communications, and, in developing hyperlocal weather forecast models.

The observed differences in WBGT between weather stations appear to be forced, in part, by differences in both air temperatures and dewpoint temperatures within respective marathon years, however there were not clear consistencies between stations across the marathon years. In 2023 and 2024, differences in dewpoint temperatures were more significant than that of air temperature, while in 2022 the opposite was observed. Generally, across both frameworks, AS15 and AS18 had among the highest WBGT conditions, on average, along with the highest air temperatures. Such differences suggest no one variable is predominantly responsible for explaining spatial differences in WBGT. While it may be reasonable to hypothesize the stations closest to Lake Michigan would exhibit the highest dewpoint temperatures and potentially the coolest air temperatures, based on the potential for advection of cool and moist air in from the lake, this was not consistently the case. Thermodynamically, a land-breeze would be anticipated for a near-coastal location in the morning hours as radiative cooling of the land surface would favor offshore flow (Keeler and Kristovich 2012; Chakraborty et al. 2023). A cooling lake-breeze would be more prominent in the afternoon hours, after which time the AS5 would have likely closed following all runners completing this segment of the course. Examining the timeseries of WBGT for the FC station, the other location near the lake, a relatively sudden but maintained decrease in WBGT is evident at the FC station in the early afternoon each year consistent with a potential onshore wind flow. Here, the FC station reports the entire race, thus the period is sufficient to capture a potential lake-breeze against the diurnal increase in temperature and within the micro-scale variations in turbulent flow and cloud cover. While a lack of wind direction data within the observations inhibits a full analysis here, evidence from 2023 – for which wind direction data are available – suggests a plausible association. In 2023, wind directions in the respective sampling periods did not meaningfully change for AS5-18, while wind direction at FC did shift from a general southwesterly flow to southeasterly and eventually easterly in the early afternoon before reverting to more south-southwesterly flow (Supplemental Fig. 9). The timing of this wind direction change however does not precisely align with the timing of changes in WBGT suggesting additional mechanisms are involved, potentially associated with the recording of light drizzle at the FC station at approximately 2:05pm, where wetting of the globe could reduce the reading and decrease the WBGT. Future research into the dynamical and thermodynamical processes that result in the observed spatial variations in WBGT, air temperature, and dewpoint temperature is warranted in order to more fully explain why these differences were observed and how that information could be applied to enhance the operational management and weather forecasting of the Chicago Marathon.

In comparing station results to a gridded product of WBGT, ERA5-Land underrepresents WBGT conditions by an average of 1.7 °C across all stations and marathon years. The bias was larger on days with higher temperatures and less persistent cloud cover (e.g., 2022: -3.1 °C) and substantially less of an underestimation on days with more consistent cloud cover (e.g., 2023; -0.7 °C), suggesting the model algorithm is insufficiently representing solar radiation. Further, the bias was largest for grid cells located on the eastern portion of the domain and is partially attributed to those grid cells containing portions of Lake Michigan land cover at the sub grid cell level. For example, the grid cell aligned with FC and AS5 has approximately 70% lake landcover which would fundamentally alter estimated WBGT relative to a grid cell with 100% urban or land cover. Underestimation of heat stress conditions from ERA5-Land relative to observations aligns with prior research within urban areas (e.g., Yu et al. 2026), and with sources of potential error in model algorithms for cloud cover, sea / lake-breeze dynamics, and coastline approximation (e.g., Ahn et al. 2024). Prior research into the efficacy of ERA5-Land for heat stress studies are mixed, where studies such as Thompson and Brown (2026) concluded commensurate estimates of WBGT between ERA5-Land and the US Climate Reference Network. We attribute the differences between studies largely due to the fact that the Thompson and Brown (2026) study, and others (e.g., Lai and Koh 2026), are comparing modeled WBGT estimates to WBGT estimates calculated from observations instead of, here, in which direct WBGT observations are compared to model WBGT estimates and further the misalignment of land covers at the sub-grid cell scale of ERA5-Land relative to the station locations.

### Limitations

The primary limitation of the Kestrel 5400 device is its increased WBGT bias during WBGT conditions of 32 °C (0.7°-0.9 °C) or higher, which may lead to a higher WBGT flag level (Clark and Konrad 2024). However, these temperatures are rare in Chicago in October (MRCC 2025). As such there is confidence in the ability of the Kestrel 5400 to accurately measure WBGT for the Chicago Marathon as currently scheduled, but further testing would be necessary to assess these results for different events and times of year. While stations were calibrated and compared to each other for consistency in advance of deployment, it is possible for there to be differences in inter-unit accuracy, where the 1.5 m height above ground level is largely indicative of average runner chest height but acknowledging variability among racers’ height exist. Inherent to the study design, temporal autocorrelation of samples is likely, necessitating the use of a Welch’s ANOVA with Games-Howell Post Hoc tests. The three-year period of record further represents a relatively small sample of events. Despite these potential limitations, results suggest a robust signal such that there is confidence in the conclusions, which may be applied to operations of the marathon. Finally, the Rolling Intervals framework is inherently measuring two types of variations: spatial variability and diurnal variability. It is not logistically feasible to separate these two components within the observations, but its inclusion here provides key context to the exposure of the runners to environmental conditions.

## Conclusions

The purpose of this research was to assess if significant differences in WBGT, and related meteorological conditions, occur spatially across the Chicago Marathon course. Using a micro-network of five observation stations deployed during the 2022, 2023, and 2024 Chicago Marathons, statistically significant differences in WBGT, air temperature, dewpoint temperature, Heat Index, and Humidex were observed across the course during all three marathons using two different analysis frameworks (*p* < 0.01). Results indicate a practical difference in WBGT for operational management, with maximum spatial differences in WBGT of 9.3 °C and thus sufficient for resulting in meaningfully different WBGT flag levels across the course upwards of 38% of the time. Observations of meteorological conditions and metrics of heat stress, such as WBGT, at multiple locations throughout a course appear warranted to appropriately capture meteorological variability and should be considered by marathon managers.

## Supplementary Information

Below is the link to the electronic supplementary material.

Supplementary Material 1

## Data Availability

The datasets used for this study are proprietary information and thus not publicly available. Requests for data access can be made by contacting the WKU Disaster Science Operations Center at DSOC@wku.edu. Observations of meteorological conditions for the Midway Airport in Chicago, used for informal comparisons to station data, were accessed and publicly available from the Iowa Environmental Mesonet at https://mesonet.agron.iastate.edu/request/download.phtml?network=IL_ASOS. Gridded estimates of WBGT from ERA5-Land are available publicly at the Copernicus Climate Data Store (https://cds.climate.copernicus.eu/datasets/reanalysis-era5-land?tab=download).
