# Supplementary material for: Spatial variations in wet-bulb globe temperature during the Bank​ of America Chicago Marathon
Source: Int J Biometeorol. 2026 Nov 2;70(11):292. doi: 10.1007/s00484-026-03361-8 (PMC13727811; doi:10.1007/s00484-026-03361-8)
Supplement: Supplementary file 1 [file 484_2026_3361_MOESM1_ESM.docx]

**Supplemental Material**

**Supplemental Figure 1**. Location and meteorological setup of the Kestrel weather station at AS5.

**Supplemental Figure 2**. As in Supplemental Figure 1, but for AS11.

**Supplemental Figure 3**. As in Supplemental Figure 1, but for AS15.

**Supplemental Figure 4**. As in Supplemental Figure 1, but for AS18.

**Supplemental Figure 5**. As in Supplemental Figure 1, but for FC.

**Supplemental Figure 6**. Time series of air temperature at AS5 (solid gray), AS11 (solid black), AS15 (dashed gray), AS18 (dashed black), and FC (dotted black) weather stations in °C for the (a) 2022, (b) 2023, and (c) 2024 Chicago Marathons. Red dots represent hourly observations from the Chicago Midway ASOS station.

**Supplemental Figure 7**. As in Supplemental Figure 6, but for dewpoint temperature.

**Supplemental Figure 8.** 7:30-11:00 am time series of Humidex (a, c, e) and Heat Index (b, d, f) at AS5 (solid gray), AS11 (solid black), AS15 (dashed gray), AS18 (dashed black), and FC (dotted black) weather stations in °C for the (a, b) 2022, (c, d) 2023, and (e, f) 2024 Chicago Marathons.

**Supplemental Figure 9**. 2023 wind speed directions, averaged into 30-minute bins for ease of interpretation, for the five weather stations from 7:30 to 3:00 local time.
